# Expanding access to early phase trials: the CATCH-UP.2020 experience

**DOI:** 10.1093/jncics/pkac087

**Published:** 2022-12-16

**Authors:** Joaquina C Baranda, Francisco J Diaz, Larry Rubinstein, Anthony F Shields, Farshid Dayyani, Amitkumar Mehta, Janice M Mehnert, Jonathan Trent, Rodwell Mabaera, Margaret Mooney, Jeffrey A Moscow, James Doroshow, Brittany Waters, Percy Ivy, Steven D Gore, Alexandra Thomas

**Affiliations:** Department of Medical Oncology, University of Kansas Cancer Center, Kansas City, KS, USA; Department of Biostatistics & Data Science, The University of Kansas Medical Center, Kansas City, KS, USA; National Cancer Institute, Division of Cancer Treatment and Diagnosis, Cancer Therapy Evaluation Program, Investigational Drug Branch, Bethesda, MD, USA; Department of Oncology, Karmanos Cancer Institute, Wayne State University, Detroit, MI, USA; Division of Hematology/Oncology, Department of Internal Medicine, Chao Family Comprehensive Cancer Center-University of California Irvine, Irvine, CA, USA; Division of Hematology and Oncology, Department of Internal Medicine, O’Neal Comprehensive Cancer Center-University of Alabama at Birmingham, Birmingham, AL, USA; Department of Internal Medicine, Perlmutter Cancer Center of NYU Langone, NYU Grossman School of Medicine, New York, NY, USA; Department of Internal Medicine, Sylvester Comprehensive Cancer Center, University of Miami, Miller School of Medicine, Miami, FL, USA; Section of Medical Oncology, Norris Cotton Cancer Center, Geisel School of Medicine at Dartmouth, Lebanon, NH, USA; National Cancer Institute, Division of Cancer Treatment and Diagnosis, Cancer Therapy Evaluation Program, Investigational Drug Branch, Bethesda, MD, USA; National Cancer Institute, Division of Cancer Treatment and Diagnosis, Cancer Therapy Evaluation Program, Investigational Drug Branch, Bethesda, MD, USA; National Cancer Institute, Division of Cancer Treatment and Diagnosis, Cancer Therapy Evaluation Program, Investigational Drug Branch, Bethesda, MD, USA; National Cancer Institute, Division of Cancer Treatment and Diagnosis, Cancer Therapy Evaluation Program, Investigational Drug Branch, Bethesda, MD, USA; National Cancer Institute, Division of Cancer Treatment and Diagnosis, Cancer Therapy Evaluation Program, Investigational Drug Branch, Bethesda, MD, USA; National Cancer Institute, Division of Cancer Treatment and Diagnosis, Cancer Therapy Evaluation Program, Investigational Drug Branch, Bethesda, MD, USA; Department of Internal Medicine, Atrium Health Wake Forest Baptist Comprehensive Cancer Center, Winston-Salem, NC, USA

## Abstract

**Background:**

Disparities in cancer outcomes persist for underserved populations; one important aspect of this is limited access to promising early phase clinical trials. To address this, the National Cancer Institute–funded Create Access to Targeted Cancer Therapy for Underserved Populations (CATCH-UP.2020) was created. We report the tools developed and accrual metrics of the initial year of CATCH-UP.2020 with a focus on racial, ethnic, geographic, and socioeconomically underserved populations.

**Methods:**

CATCH-UP.2020 is a P30 supplement awarded to 8 National Cancer Institute–designated cancer centers with existing resources to rapidly open and accrue to Experimental Therapeutics Clinical Trials Network (ETCTN) trials with emphasis on engaging patients from underserved populations. Sites used patient-based, community-based, investigator-based, and program-based tools to meet specific program goals.

**Results:**

From September 2020 to August 2021, CATCH-UP.2020 sites opened 45 ETCTN trials. Weighted average trial activation time for the 7 sites reporting this was 107 days. In the initial year, sites enrolled 145 patients in CATCH-UP.2020 with 68 (46.9%) representing racial, ethnic, rural, and socioeconomically underserved populations using the broader definition of underserved encompassed in the grant charge. During the initial year of CATCH-UP.2020, a time impacted by the COVID-19 pandemic, 15.8% (66 of 417) and 21.4% (31 of 145) of patients enrolled to ETCTN trials at network and at CATCH-UP sites, respectively, were from racial and ethnic minority groups, a more limited definition of underserved for which comparable data are available.

**Conclusion:**

Targeted funding accelerated activation and accrual of early phase trials and expanded access to this therapeutic option for underserved populations.

Amid immense innovation in cancer care, large-scale disparities in cancer outcomes remain a challenge (1-6). These stark disparities are multifactorial and impact a variety of underserved populations, with disparate outcomes observed among racial and ethnic groups, by socioeconomic status and by geographic location (1,7-11). The often-limited representation of underserved populations in clinical trials (12-16) is a critical factor contributing to disparities. In 2020, the US Food and Drug Administration’s Center for Drug Evaluation and Research reported that Black patients represented only 8% of the 32 000 patients who participated in the trials leading to the approval of 53 novel drugs (17). Reported barriers to participation of Black patients in clinical trials include lack of awareness, economic hurdles, communication challenges, and mistrust (18), as well as trial-specific eligibility criteria (19,20).

Patients from lower socioeconomic groups and those living in geographically isolated areas also experience inequities in cancer outcome (21). Death from colorectal cancer for men living in the poorest counties in the United States is 35% higher than for men from the most affluent counties (1,8). These differences may be attributed to lack of health insurance coverage, barriers to early detection, mistrust, and inequity in access to new treatments associated with improved outcome, including treatments on clinical trials (22). Misconceptions about and limited awareness of clinical trials likely impact both rural and lower socioeconomic group patients (23).

Differences in cancer risk and biology in underserved populations may also contribute to outcomes disparities, particularly if these groups are underrepresented in clinical trials as the impact of newer therapies could be inadequately studied in these populations (23-28). For example, recent biomarker analyses from the Carolina Breast Cancer Study demonstrated that Black women more frequently have higher-risk, harder to treat, hormone receptor–positive breast cancer than women of other racial groups (29). Clinical trial populations that differ from treatment populations limit generalizability of trial results and threaten to perpetuate outcome disparities (30,31).

Clinical trial participation correlates with reduction in mortality (32). Evidence supports that access to clinical trials is also a vital component of addressing outcome disparities with a report from the SWOG Cancer Research Network demonstrating that access to clinical trials narrows the gap in cancer care disparity among patients in urban and rural communities (33). Direct benefit from participation in early phase trials was addressed by the American Society of Clinical Oncology in their 2017 policy statement on phase I trials; this reaffirmed the critical role of early phase trials in cancer research and treatment and reviewed the evidence that early phase trial patients may achieve improvement in quality of life and experience psychological as well as direct medical benefit (34).

To expand access and increase accrual of underserved populations into early phase trials, a congressional mandate introduced by Senator Richard Shelby of Alabama funded the Create Access to Targeted Cancer Therapy for Underserved Populations, CATCH-UP.2020, (hereafter CATCH-UP). CATCH-UP was designed to respond to the ethical, clinical, and public mandate to eliminate disparities in cancer research and outcomes. Eight National Cancer Institute (NCI)–designated cancer centers that demonstrated robust ability to accrue minority and underserved populations to early phase clinical trials but were not members of the Experimental Therapeutics Clinical Trials Network’s (ETCTN) UM1 program were selected to participate. Here, we report the tools developed and accrual metrics of the initial year of CATCH-UP with a focus on racial, ethnic, geographic, and socioeconomically underserved populations.

## Methods

### Site selection

CATCH-UP is a P30 administrative supplement award to enhance access to targeted cancer therapy for minority and underserved populations to ETCTN trials. The sites were selected using a peer review process similar to that of a special interest panel. Qualified reviewers were chosen and assigned proposals as primary or secondary reviewers. There was a minimum of 3-4 reviewers. The applications were scored using the NCI scale. The scores were averaged, and the median and mean calculated. Each application was ranked based on the mean score with a review of the standard deviation also considered based on a highly constrained sample size. The special interest panel met and discussed the applications. Reviewers were given the opportunity to revise their scores. A funding line was determined based on available funding. Investigators were notified if their application was recommended for funding. The funding plan was presented to senior leadership for the next level of review. Once the funding plan was approved, the investigators were notified if their application was approved for funding. Eight NCI-designated cancer centers were selected to receive this supplement to their Cancer Center Support Grant: Atrium Health Wake Forest Baptist Comprehensive Cancer Center, Chao Family Comprehensive Cancer Center–University of California Irvine, Dartmouth-Hitchcock Norris Cotton Cancer Center, Karmanos Cancer Institute–Wayne State University, O’Neal Comprehensive Cancer Center–University of Alabama at Birmingham, Perlmutter Cancer Center of New York University Langone Health, Sylvester Comprehensive Cancer Center-University of Miami Health System, and University of Kansas Cancer Center.

Successful applicants for CATCH-UP demonstrated established clinical outreach programs in underserved racial, ethnic, rural, or socioeconomically underserved populations and infrastructure for participation in ETCTN trials. Sites were charged with rapidly activating ETCTN trials and engaging minority and underserved populations with the goal of improving minority and underserved patient participation in these trials, which test novel cancer therapies. Sustainability and resource availability were key considerations, and sites were required to have access to genomic testing capabilities, community outreach offices, and clinics as well as a catchment area with a substantial proportion of minority and underserved patients based on race, ethnicity, rural, and/or low-income area residency.

For this initiative, minority and underserved populations include members of minority racial and ethnic groups or other individuals experiencing disparities including refugees, individuals with limited English proficiency, individuals with disabilities, sexual gender minorities, social-economically disadvantaged populations, people living in a geographic area with a shortage of health-care services, and groups that face economic barriers to health care.

In September 2020, the CATCH-UP grant was activated for a 1-year project period. The requirements for each site included accrual of a minimum of 24 patients to ETCTN trials with approximately 50% of these patients representing minority and underserved populations.

The CATCH-UP sites selected have experimental therapeutics programs and community outreach programs. This project was led by an oncologist with experience in early phase trials and by an investigator with community engagement expertise at each site. Disease-focused clinical investigators in oncology subspecialties were included to work with program leaders as site investigators for disease-specific trials and to engage eligible patients. In addition, early career investigators were paired with disease-focused clinical investigator mentors to participate in the conduct of ETCTN trials and to take part in ETCTN meetings. Progress reports were presented during monthly video-conference meetings, which included all CATCH-UP sites and NCI program leadership. Awardees were also encouraged to participate in ETCTN disease-specific meetings. Annual reports were included in individual NCI Cancer Center Support Grant progress reports.

### CATCH-UP operations

The sites were offered selected ETCTN trials; each trial required a protocol amendment to enable CATCH-UP participation. At CATCH-UP sites, existing regulatory processes were reviewed and aligned to prioritize activation of these trials. Built on existing resources, each site developed and shared best practices for using patient-based, community-based, investigator- and program-based tools to address the charge of CATCH-UP (Table 1). Additional support from site cancer center leadership, local government, and philanthropic sources at several sites provided coverage for the research-related tumor biopsies and other study-related costs.

**Table 1.** Tools used to enhance accrual to ETCTN trials with a focus on underserved populations^a^

Focus area	Tools
Patient based	Patient health navigation—nonclinical navigators who are racially, geographically concordant who facilitate patient interaction with health-care systems with a focus on clinical trials Telehealth option for initial screening visits, remote consenting, and follow-up as appropriate with increased focus on underserved minorities Engagement of patient advocates to identify barriers to minority accrual to CATCH-UP Systematic review of trials to reduce patient inconvenience Financial counseling Availability of interpreters (preferably in person) to review study documents, consent, and address questions Expanding accepted payors, such as Medicaid and other nonprivate programs
Community based	Selected trials activated at outreach sites, including focused selection of outreach sites with large, underserved populations For trials that could not be activated locally, co-management with outreach oncologist offered (ie, patients are seen at main center for experimental therapy on day 1 of a 21-day cycle but can receive day 8 and day 15 therapy, which does not include an experimental component at affiliated outreach site) Education and training of outreach clinical staff on CATCH-UP and the importance of engaging underserved patient populations Press release on the award Social media used to raise awareness of ETCTN trial availability
Investigator based	Regular newsletter to internal and outreach providers Regular (at least monthly) site meetings on CATCH-UP Clinical trials application to search for trials (available to providers and patients) Recognition of high accruing investigators Disease-site groups selected ETCTN trials based on catchment area needs and trial portfolio Engagement of subspecialties as subinvestigators such as interventional radiologists for quality tissue acquisition and rheumatologists, gastroenterologists, dermatologists, and neurologists included for toxicity management
Program based	CATCH-UP navigator or project manager identified at sites Monthly calls with NCI program leadership NCI program leadership facilitated resolution to trial-specific issues Funding from other sources (cancer center, local government, philanthropic) to support research staff and study procedures CATCH-UP core group formed to modify clinical trial workflow to accelerate trial activation CATCH-UP site participation in NCI ETCTN disease-focused meetings

a Tools used by some but not all of the CATCH-UP sites. CATCH-UP = Create Access to Targeted Cancer Therapy for Underserved Populations; ETCTN = Experimental Therapeutics Clinical Trial Network; NCI = National Cancer Institute.

Patients were identified as representing underserved communities if they self-identified into these groups or their identity was clear to the provider: racial and ethnic minorities, refugees, individuals with limited English proficiency, and individuals with disabilities. Federal and state agency definitions were used to identify social and economically disadvantaged populations, as well as people living in an underserved geographic area (eg, rural or frontier areas) and those areas with a shortage of health-care services for primary care and groups that face economic barriers to health care. Specific tools used were rural-urban commuting area codes (rural defined as rural-urban commuting area code ≥ 4), rural-urban continuum codes (rural defined as rural-urban continuum codes 4-9), state-level agency definitions of rural populations and the Health Resource and Services Administration database on geographic and population Health Professional Shortage Area for primary care.

### Statistical analysis

In September 2021, the 8 sites participating in the CATCH-UP program were asked to submit accrual reports by month between September 1, 2020, and August 31, 2021. In addition to the total accrual, the total minority accrual and the frequencies of Black, Hispanics, Asian or Pacific Islanders, Native Americans patients; patients from rural areas; patients of low income and/or Health Professional Shortage Area; and “others” not included in these categories were reported including patients with disabilities. The 8 sites completed the report.

For comparison purposes, accrual frequencies for the ETCTN network sites were obtained from a dataset provided by the NCI. Regarding underserved populations, the ETCTN dataset included only information on racial and ethnic minorities, as rural and socioeconomic status reporting has not been required across the broader network.

For CATCH-UP sites and ETCTN sites, proportions of accrued racial and ethnic minorities were calculated along with their 95% logit-transformed confidence intervals for the time periods, September 1, 2020, to March 31, 2021 (CATCH-UP start-up period) and April 1, 2021, to August 31, 2021 (CATCH-UP period). Of the analyzed ETCTN accrued patients, 4% had an unknown or unreported ethnic or racial status. These were imputed as nonminority for these analyses.

The 8 CATCH-UP sites provided the number of trials that were opened in the above period. Of these sites, 7 provided the average number of days to trial activation. Because of severe staffing issues and closure of their clinical research operations during the COVID-19 pandemic, 1 site was unable to provide data on time to trial activation during the initial year of CATCH-UP.

The average number of days to CATCH-UP trial activation was estimated as the average of the averages provided by the sites, weighted by the number of trials opened. Statistical analyses were conducted with Stata 15.1 (StataCorp LLC, College Station, TX, USA).

## Results

Overall, 45 ETCTN trials using NCI investigational new drug agents in areas of unmet medical needs were opened across the 8 CATCH-UP sites (Table 2). More than 40% of these trials were phase I or phase I and II. The average number of CATCH-UP sites that participated in each of these trials was 2.8. The number of ETCTN trials opened in each site ranged from 13 to 24 trials with median of 15.5. The weighted average of time to trial activation was 107 days (minimum site average = 44 days, maximum = 171 days, n = 7 sites). For these 45 trials, the average overall study-wide planned accrual was 68 patients (range = 15-312) with an average overall actual study-wide accrual of 34 (range = 2-143) patients as of July 25, 2022. Study treatment included single agent or combination therapies utilizing chemotherapy, immunotherapy, small molecule kinase inhibitors, antibody-drug conjugates, and other novel agents. In addition to systemic therapies, 6 trials included radiation therapy and 2 trials with radiopharmaceuticals. Some of the trials required biopsies at screening and on treatment. On October 16, 2020, the first patient was enrolled in this program. In December 2020, all 8 centers had trials activated, and each site had accrued patients by April 2021. Characteristics of patients enrolled in ETCTN studies at CATCH-UP sites by underserved status are presented in Table 3.

**Table 2.** Study phase and number of activated CATCH-UP sites for 45 ETCTN studies

Characteristic	No. of studies	Proportion, % (of 45 studies)
Phase		
I	8	17.8
I/II	11	24.4
II	26	57.8
No. of CATCH-UP sites at which a study was activated^a^		
1	8	17.8
2	16	35.6
3	8	17.8
4	7	15.6
5	4	8.9
6	1	2.2
7	1	2.2

a The average number of CATCH-UP sites per ETCTN study was 2.8 (95% CI = 2.3 to 3.2). CATCH-UP = Create Access to Targeted Cancer Therapy for Underserved Populations; CI = confidence interval; ETCTN = Experimental Therapeutics Clinical Trial Network.

**Table 3.** Characteristics of patients enrolled in CATCH-UP studies

Variables	Not underserved (n = 77)	Underserved (n = 68)	All (n = 145)
Mean age (95% CI), y	66.0 (63.4 to 68.7)	61.6 (58.6 to 64.6)^a^	64.0 (62.0 to 66.0)
Sex, % (No)			
Female	62.3 (48)	75.0 (51)	68.3 (99)
Male	37.7 (29)	25.0 (17)	31.7 (46)
Disease stage, % (No.)			
Metastatic	92.2 (71)	86.8 (59)	89.7 (130)
Regional	7.8 (6)	13.2 (9)	10.3 (15)
Study site, % (No.)			
KCI	15.6 (12)	8.8 (6)	12.4 (18)
KUCC	28.6 (22)	36.8 (25)	32.4 (47)
MSCC	5.2 (4)	8.8 (6)	6.9 (10)
NCCC	0.0 (0)	10.3 (7)	4.8 (7)
NYU	6.5 (5)	4.4 (3)	5.5 (8)
UAB	10.4 (8)	4.4 (3)	7.6 (11)
UCI	10.4 (8)	7.4 (5)	9.0 (13)
WF	23.4 (18)	19.1 (13)	21.4 (31)

a Age was not available from 1 underserved patient. CATCH-UP = Create Access to Targeted Cancer Therapy for Underserved Populations; CI = confidence interval; KCI = Karmanos Cancer Institute–Wayne State University; KUCC = University of Kansas Cancer Center; MSCC = Sylvester Comprehensive Cancer Center–University of Miami Health System; NCCC = Dartmouth-Hitchcock Norris Cotton Cancer Center; NYU = Perlmutter Cancer Center of New York University Langone Health; UAB = O’Neal Comprehensive Cancer Center–University of Alabama at Birmingham; UCI = Chao Family Comprehensive Cancer Center–University of California Irvine; WF = Atrium Health Wake Forest Baptist Comprehensive Cancer Center.

In the initial year of CATCH-UP, the 8 sites accrued 145 patients to ETCTN trials, of whom 68 (48.6%) patients were from underserved populations. Of the 145 patients, 31 (21.4%) were from racial and ethnic minorities, 18 (12.4%) represented rural underserved populations, and 32 (22.1%) represented socioeconomically underserved populations that were encompassed in the broader scope of CATCH-UP.

The comparison of CATCH-UP site underserved accrual to other ETCTN sites is complicated by the lack of data regarding ETCTN accrual of rural and economically underserved populations, even though these populations substantially contribute to the CATCH-UP underserved accrual (30.3%). Accrual of racial and ethnic minorities in ETCTN sites was compared with that of CATCH-UP sites during the grant period (Table 4). Of the 68 individual underserved patients, 15 represented more than 1 underserved category (Table 5). For the first year of CATCH-UP, patients from racial and ethnic minority groups represented 15.8% (66 of 417) and 21.4% (31 of 145) of patients enrolled at ETCTN sites and CATCH-UP sites, respectively.

**Table 4.** Racial and ethnic minority accrual to ETCTN trials at network and CATCH-UP sites by time period

Accrual type^a^	Time period			
	Start-up period: September 1, 2020 - March 31, 2021		CATCH-UP period: April 1, 2021 - August 31, 2021	
	No.	% (95% CI)	No.	% (95% CI)
ETCTN sites				
Nonminority accrual^b^	213^c^	85.5 (80.6 to 89.4)	138^d^	82.1 (75.6 to 87.2)
Minority accrual	36	14.5 (10.6 to 19.4)	30	17.9 (12.8 to 24.4)
CATCH-UP sites				
Nonminority accrual	52	77.6 (65.9 to 86.2)	62	79.5 (68.9 to 87.1)
Minority accrual	15	22.4 (13.8 to 34.1)	16	20.5 (12.9 to 31.1)

a Reports accrual of racial and ethnic minorities including Black or African American, American Indian, or Alaska Native, Asian or Pacific Islander, and Hispanic or Latino. CATCH-UP = Create Access to Targeted Cancer Therapy for Underserved Populations; CI = confidence interval; ETCTN = Experimental Therapeutics Clinical Trial Network.

b Patients from ETCTN sites who had unknown or unreported ethnic or racial status were classified as nonminority for these conservative data analyses.

c During the start-up period, there were 10 patients with unknown or unreported racial or ethnic status in the ETCTN sites.

d During the CATCH-UP-period, there were 9 patients with unknown or unreported racial or ethnic status in the ETCTN sites.

**Table 5.** Frequencies for the 68 underserved patients accrued to ETCTN trials at CATCH-UP sites in initial funding year^a^

Second Race (ethnicity)	Non-low income and non-HPSA		Low income and/or HPSA	
	Nonrural	Rural	Nonrural	Rural
Asian and Pacific Islander (non-Hispanic)	3	—	3^b^	—
Black (Hispanic)	1	—	—	—
Black (non-Hispanic)	14	1	3^c^	—
White (Hispanic)	4	—	2	—
White (non-Hispanic)	2^d^	11	18	6

a The table does not include 77 accrued patients who were not classified as underserved. “—” signifies no patients in this category were identified. CATCH-UP = Create Access to Targeted Cancer Therapy for Underserved Populations; ETCTN = Experimental Therapeutics Clinical Trial Network; HPSA = Health Professional Shortage Area.

b Of the 3 patients, 2 have disabilities.

c Of the 3 patients, 1 has a disability.

d The 2 patients have disabilities.

## Discussion

CATCH-UP is a unique initiative to expand access to cutting-edge therapy through enrollment in early phase clinical trials. Notably, the challenge of engaging minority and underserved populations is profound in early phase trials, which are generally complex and often accessible only at larger centers in metropolitan areas. This project also had a short execution timeline. CATCH-UP sites used a variety of tools, many of which extended across sites, and others adapted to be more site specific, to increase engagement and accrual of patients from minority and underserved populations to clinical trials.

Conducted during the COVID-19 pandemic, the average time to trial activation seen at CATCH-UP sites of 107 days was relatively rapid. A recent program dedicated to accelerating trial activation at an NCI-designated cancer center successfully decreased trial activation time from a median of 185 days to a median of 132 days (35). Notably this work was across a portfolio of trials in contrast CATCH-UP focuses on often complex, federally funded early phase trials.

Many of the CATCH-UP sites learned and shared best practices for outreach to underserved communities, including coordination with distant outlying sites, integration of telemedicine, and other technology for precision medicine, and developed a road map for comprehensive centers to further extend access to clinical trials to their full catchment areas. These practices included trial selection, use of population health navigators, community-based investigators, use of telemedicine, and screening of genomic data. Although some of these tools, such as telemedicine, have been previously available, substantial short-term funding markedly facilitated their utilization for the goals of CATCH-UP. Key to successful accrual in some sites was additional funding from other resources to cover research-related procedures. Despite these tools, CATCH-UP sites observed only modestly higher accrual of racial and ethnic minorities than that observed at ETCTN sites. As a 1-year program, the CATCH-UP sites faced short timelines. Many of the CATCH-UP sites activated ETCTN trials for the first time, however, most of the ETCTN trials had already been activated and actively accruing at ETCTN sites. Barriers inherent to early phase trial accrual included travel distance for patients; time commitment of patients and caregiver; slot availability; delays in availability of laboratory kits; and COVID-19 infection in patients, caregivers, and staff.

The CATCH-UP program allowed engagement and accrual of underserved populations in broader terms than generally systematically reported in clinical trials. Of the 145 patients, 44 (30.3%) accrued were from rural or socioeconomically underserved settings. Although direct comparison with historical controls for this subgroup is not readily available, multiple reports have found structural challenges associated with engaging these populations around trial participation (36-39). Several models used, such as co-management of patients with local oncologists, required initial infrastructure support but will require less long-term funding, suggesting that aspects of this work could be sustainable and expanded to a broader portfolio of trials. Accrual of patients from racial and ethnic minority populations, although higher than that of ETCTN sites, was not markedly more at CATCH-UP sites. Possible reasons for this include that the data presented here reflect the early stages of a program and that more time will allow for consistent implementation of the tools developed. For example, co-management with local providers requires completion of regulatory processes and was often not enabled until the later months of CATCH-UP. Additionally, the portfolio of trials offered through ETCTN during this shorter initial time period may have had an impact. For example, limited trial options were available for colon cancer and gastric cancer, diseases that disproportionately impact Black and Hispanic populations, respectively (40). This could become more balanced over time.

With short-term funding, monitoring and the focused selection of sites with underserved populations, CATCH-UP sites, in an initial year, which was also complicated by an unprecedented pandemic, were able to consistently accrue higher portions of minority and underserved patients than network sites. Sustaining this effort, which may require less intense resource allocation over the long term, could allow sites to truly expand access and have trial populations more reflective of catchment area populations. A period with less impact of COVID-19 on the health-care system may allow more complete evaluation of the tools used in this program. Additionally, opportunities remain to cross-fertilize tools developed to engage underserved populations at the CATCH-UP sites with other ETCTN sites.

Importantly, health-care delivery strategies that emerged or became more facile during the early months of the COVID-19 pandemic may inform our approach to improving access for minority and underserved populations. Considerations included changes in study monitoring, use of alternative methods for safety assessments such as phone contact, virtual visit, and alternative locations for assessment including laboratory tests and imaging. Remote consenting and telehealth visits were adapted in the informed consent process and safety assessments when appropriate. These modalities were used by CATCH-UP sites, and ongoing use could offer sustained increases in access to trials. Regulations that disallow the use of telehealth across state borders and the digital divide, which often impacts underserved populations (41,42), are ongoing challenges to sustained widespread use of these technological tools to overcome access barriers.

Future opportunities for this work include sustaining these efforts at a lower cost and further cross-fertilization of successful tools among CATCH-UP sites and across the ETCTN network. Partnering with community oncologists in a consistent manner around trial access for underserved populations, a process that CATCH-UP has started, remains a promising area in need of sustainable infrastructure. Finally, opportunities remain to further expand inclusivity such as providing access to veterans, many of whom are from underserved populations.

At this early juncture, and during an unprecedented pandemic, the resources and tools used in CATCH-UP have expanded access to early phase trials for patients from minority and underserved populations. Further refinement and expansion of these tools could further broaden access to early phase trials, as well as to trials across the spectrum of investigation, with the goals of overcoming cancer disparities and improving cancer outcomes.

## Data Availability

Detailed data cannot be shared publicly to protect the privacy of individual clinical trial participants. Information to support the findings of these analyses are available by contacting the corresponding author, who upon reasonable request and understanding of the intended use of the data will work with the Clinical Trials Evaluation Program to provide the requested information in the manner which continues to protect individual patient information. Data generated by these analyses are provided in the manuscript.

## Notes

**Role of the funder:** In this NCI-funded program, employees of the funding agency developed the initial concept for the program, provided data, and contributed to the preparation of the manuscript.

**Disclosures:** JB Research Funding (to the institution): Sanofi, Pfizer, Astellas, Xencor, Impact Therapeutics, Synermore Biologics, Changchun Intellicrown Pharm, Exelixis, Tolero, SQZ, Nektar, Takeda; Consulting: Sanofi; Stock ownership: Forty-seven, Moderna, Zymeworks, Aprea, Merus, Hylapharm. FD Research Support (to the institution): Amgen, AstraZeneca, Bristol-Myers Squibb, Merck, Genentech/Roche, Taiho, Exelixis, Trishula, Leap Therapeutics; Consulting Eisai, Ipsen, Exelixis, Sirtex, Deciphera, Ipsen, Natera, and Genentech/Roche. AT Research Support (to the institution): Sanofi, Merck; Stock ownership: Johnson and Johnson, Bristol Myers Squibb, Pfizer, Gilead, Doximity; DSMB: BeyondSpring Consulting: Lilly, Genentech; Royalties: Up-to-Date. All other authors report no conflicts.

**Author contributions:** Jeffrey A. Moscow, MD (Conceptualization; Resources; Supervision; Writing—review & editing), James Doroshow, MD (Funding acquisition; Resources; Writing—review & editing), Rodwell Mabaera, MD (Data curation; Funding acquisition; Resources; Writing—review & editing), Margaret Mooney, MD, MS (Funding acquisition; Resources; Writing—review & editing), Steven D. Gore, MD (Conceptualization; Data curation; Funding acquisition; Resources; Supervision; Writing—review & editing), Alexandra Thomas, MD (Conceptualization; Data curation; Funding acquisition; Resources; Supervision; Writing—original draft; Writing—review & editing), Brittany Waters, BS (Funding acquisition; Resources; Writing—review & editing), Percy Ivy, MD (Conceptualization; Data curation; Funding acquisition; Resources; Supervision; Writing—review & editing), Larry Rubinstein, PhD (Conceptualization; Data curation; Formal analysis; Writing—review & editing), Anthony F. Shields, MD (Funding acquisition; Resources; Writing—review & editing), Joaquina C. Baranda, MD (Conceptualization; Data curation; Funding acquisition; Resources; Supervision; Writing—original draft; Writing—review & editing), Francisco J. Diaz, PhD (Conceptualization; Data curation; Formal analysis; Writing—review & editing), Janice M. Mehnert, MD (Funding acquisition; Resources; Writing—review & editing), Jonathan Trent, MD (Funding acquisition; Resources; Writing—review & editing), Farshid Dayyani, MD (Funding acquisition; Resources; Writing—review & editing), Amitkumar Mehta, MD (Funding acquisition; Resources; Writing—review & editing).

**Prior presentations:** This was presented in part at the 2022 ASCO Annual Meeting, Chicago, Illinois, June 3-7, 2022.
